# Selection of N86F184D1246 haplotype of *Pfmrd1* gene by artemether–lumefantrine drug pressure on *Plasmodium falciparum* populations in Senegal

**DOI:** 10.1186/s12936-016-1490-4

**Published:** 2016-08-25

**Authors:** Aminata Mbaye, Baba Dieye, Yaye D. Ndiaye, Amy K. Bei, Affara Muna, Awa B. Deme, Mamadou S. Yade, Khadim Diongue, Amy Gaye, Ibrahima M. Ndiaye, Tolla Ndiaye, Mouhamad Sy, Mamadou A. Diallo, Aida S. Badiane, Mouhamadou Ndiaye, Mame C. Seck, Ngayo Sy, Ousmane Koita, Donald J. Krogstad, Davis Nwakanma, Daouda Ndiaye

**Affiliations:** 1 Laboratory of Parasitology/Mycology HALD, Cheikh Anta Diop University of Dakar, PO Box 5005, Dakar, Senegal; 2 Department of Immunology and Infectious Diseases, Harvard School of Public Health, Boston, MA USA; 3 Tulane University, New Orleans, LA USA; 4 Malaria Research Centre, Serrekunda, Gambia; 5 University of Bamako, Bamako, Mali

**Keywords:** *Plasmodium falciparum*, Haplotype, Artemether–lumefantrine, *pfmdr1*, Senegal

## Abstract

**Background:**

The use of artemisinin as a monotherapy resulted in the emergence of artemisinin resistance in 2005 in Southeast Asia. Monitoring of artemisinin combination therapy (ACT) is critical in order to detect and prevent the spread of resistance in endemic areas. Ex vivo studies and genotyping of molecular markers of resistance can be used as part of this routine monitoring strategy. One gene that has been associated in some ACT partner drug resistance is the *Plasmodium falciparum* multidrug resistance protein 1 (*pfmdr1*) gene. The purpose of this study was to assess the drug susceptibility of *P. falciparum* populations from Thiès, Senegal by ex vivo assay and typing molecular markers of resistance to drug components of ACT currently used for treatment.

**Methods:**

The ex vivo susceptibility of 170 *P. falciparum* isolates to chloroquine, amodiaquine, lumefantrine, artesunate, and artemether was determined using the DAPI ex vivo assay. The high resolution melting technique was used to genotype the *pfmdr1* gene at codons 86, 184 and 1246.

**Results:**

A significant decrease in IC50 values was observed between 2012 and 2013: from 13.84 to 6.484 for amodiaquine, 173.4 to 113.2 for lumefantrine, and 39.72 to 18.29 for chloroquine, respectively. Increase of the wild haplotype NYD and the decrease of the mutant haplotype NFD (79 and 62.26 %) was also observed. A correlation was observed between the wild type allele Y184 in *pfmdr1* and higher IC50 for all drugs, except amodiaquine.

**Conclusion:**

This study has shown an increase in sensitivity over the span of two transmission seasons, marked by an increase in the WT alleles at *pfmdr1*. Continuous the monitoring of the ACT used for treatment of uncomplicated malaria will be helpful.

## Background

 Among the five species of *Plasmodium* causing human malaria, *Plasmodium falciparum* is the deadliest. The latest World Malaria Report showed that of the 214 million cases of malaria recorded in 2014, 88 % were registered in sub-Saharan Africa where most of cases were caused by *P. falciparum* [1, 2].

In the late 1950s, resistance of *P. falciparum* to chloroquine (CQ) emerged in South America and Southeast Asia [3]; since then, resistance has spread rapidly around the world and in Africa the first cases were reported in 1978 [4]. The introduction of other anti-malarial drugs [sulfadoxine-pyrimethamine (SP), mefloquine (MQ), etc.] has led to the emergence of strains of *P. falciparum* that are resistant to multiple drugs in some endemic areas [5]. As a result, since April 2001, the World Health Organization (WHO) has recommended the use of artemisinin-based combination therapy (ACT) in countries where *P. falciparum* is resistant to CQ, SP, and amodiaquine (AMQ), to avoid early emergence of resistance to these molecules [6]. Despite these efforts, cases of resistance to artemisinin (ART) have emerged in Cambodia and in other regions of Southeast Asia [7]. Indeed, in these countries, ART was used as monotherapy for uncomplicated falciparum malaria. Monitoring the sensitivity of *P. falciparum* to ACT becomes essential for therapeutic management of malaria. Such monitoring can be done by various methods, including in vivo efficacy studies, in vivo clearance time assays [8], in vitro chemo-sensitivity to anti-malarial drugs (RSA), and genotyping of *pfk13* molecular markers of ART resistance [9]. For the *P. falciparum* multidrug-resistant protein 1 (*pfmdr1*) gene, several single nucleotide polymorphisms were described, but the more common were N86Y, Y184F, S1034C, N1042D, and D1246Y. Studies have shown an association of *pfmdr1* gene, specifically the codon 86, and a decrease of sensitivity to AMQ and artesunate (ARS) [10, 11]. Studies have also shown the 86Y and 1246Y mutations are strongly related to the reduction of in vivo sensitivity to artesunate-amodiaquine combination (ASAQ). Also, the N86 and D1246 alleles are related to a decrease of susceptibility to artemether–lumefantrine combination (AL) [12–14]. Moreover, the haplotype N86F184D1246 is selected by a high drug pressure of AL [15, 16], while 1034C, 1042D and 1246Y mutations have been reported to confer resistance against quinine (QN) and increased susceptibility to MQ, halofantrine (HF) and ART [17, 18].

In Senegal, ACT has been implemented as first-line treatment for uncomplicated falciparum malaria since 2006, following WHO recommendation [19]. AL and ASAQ combination are used for first-line treatment of uncomplicated *P. falciparum* malaria [2]. Ex vivo studies have been conducted to study the sensitivity of current anti-malarial drugs used against circulating *P. falciparum* populations in Senegal [20–23]. This work aims to study the consequences of use of ACT (ASAQ and AL) for uncomplicated malaria treatment in Senegal by using the ex vivo sensitivity to ARS, AMQ, artemether (AMT) and lumefantrine (LUM) of *P. falciparum* isolates from Thiès collected during the transmission period of malaria combined to the *pfmdr1* gene polymorphism at codon N86Y, N184F and D1246Y.

## Methods

### Study sites

Patient recruitment took place during the seasonal malaria period (from September to December) in Section de Lutte Anti-Parasitaire (SLAP) clinic in Thiès in 2012 and 2013. This centre has the privilege to control malaria treatment in Thiès region. The epidemiological profile of this region is characterized as sahelian with a short, seasonal transmission period, generally fewer than 4 months after the rainy season ends. The vectors found are *Anopheles arabiensis* and *Anopheles gambiae* and the entomological inoculation rate is generally low and varies from 1 year to another (0–20 infectious bites/person/year). The region of Thiès is located at 70 km from Dakar and malaria incidence in this region ranges between five and 15 per 1000 inhabitants [21, 24].

Individuals who presented at the health centre with malaria symptoms were tested by both microscopy and rapid diagnostic test (when available). The selected patients, aged from 5 to 20 years, had uncomplicated malaria due to *P. falciparum* with a parasitaemia higher than 15,000 µl. Exclusion criteria included individuals who had clinical features of severe malaria or who had a history of taking anti-malarial treatment prior to the visit. Informed consent or assent of the patient and their guardian (for children) was obtained before collecting the blood. The Human Subjects Committee of Tulane University and the Ethics Committee of the Senegal Ministry of Health in Dakar both approved the protocols used in these studies. The work was supported by the International Centres of Excellence for Malaria Research, (ICEMR) West Africa (U19AI089696).

### Blood sample collection

Venous blood was collected in EDTA tubes and filter papers were collected by finger prick. Patients were treated with AMT-LUM or ARS-AMQ combination. These samples were sent to Aristide Le Dantec Hospital within 6 h of blood draw.

### Drugs and drug preparation

AMQ hydrochloride (USP), ARS (Sigma), AMT (SIGMA), CQ diphosphate (Sigma) and LUM (USP) were used in ex vivo assays. These molecules were dissolved directly in dimethyl sulfoxide (DMSO) solvent at a concentration of 10 mM. A first dilution (1/1000) with RPMI 1640 media was done to obtain an intermediate solution. A serial dilution (1/2) was then operated to get a range of concentrations for each drug. The range of concentration varied from 100 to 0.39 nM for AMQ, 75 to 0.29 nM for ARS, 150 to 0.58 nm for AMT, 750 to 2.93 nM for CQ, and 2000 to 7.81 nM for LUM. The 96-well plates were then loaded with drugs at a final volume 20 µl per well and stored at −20 °C until use.

### Ex vivo assay

Parasitized blood samples from the field were first centrifuged to remove the plasma, then washed two times with unsupplemented media. Samples with a parasitaemia between 0.4 and 1 % were suspended in complete media, supplemented with AB serum and Albumax II to adjust the haematocrit to 2 % before being distributed into 96-well plates preloaded with drugs. If parasitaemia was higher than 1 %, a dilution with an O+ blood sample without *P. falciparum* strains was done. The plates were incubated at 37 °C under the following gas conditions (1 % O_2_, 5 % CO_2_ and 94 % N_2_) for 48 or 72 h, until parasite re-invasion as assessed by microscopy. The ex vivo response of parasites to the different anti-malarial drugs was evaluated using the DAPI molecular probe as described previously [25]. Plates were briefly thawed, spun, and re-suspended with 100 µl (3.64 nM of DAPI concentration) of DAPI-buffer and incubated for 30 min in the dark. They were washed with the PBS before reading at excitation wavelength of 358 nm and emission wavelength of 460 nm using a Fluoroskan Ascent reader. Percent growth was calculated relative to the RPMI-only control wells for each plate. The control strain used to validate results was sensitive to CQ *P. falciparum* 3D7 laboratory strain from MR4.

### DNA extraction and genotyping

The genomic DNA was extract from filter papers using the QIAmp DNA mini kit (Qiagen) following manufacturer’s instructions. The high resolution melting (HRM) technique was used for genotyping codons N86Y, Y184F and D1246Y of *pfmdr1* gene and codon K76T of *pfcrt* using specific primers and probes, as previously described [26].

### Statistical analysis

All statistical analyses were performed using Graph Pad Prism software (Version 5.0). Mann–Whitney test was used to compare the distribution of IC50 s between years. For the difference of *pfmdr1* polymorphism, the online Z test for two populations’ proportion was used. *p value* was considered significant when it was less than 0.05.

## Results

### Ex vivo susceptibility of *Plasmodium falciparum* parasites from Thiès

Among a total of 120 and 50 samples collected, respectively, in 2012 and 2013 in Thiès, 114 and 48, respectively, were tested. The eight non-tested samples had a parasitaemia lower than 15,000 parasites/µl.

Parasites’ responses to drugs at Thiès in 2012 and 2013 are listed in Table 1. Ex vivo result was validated by testing the 3D7 strain sensitive to CQ and good sensitivity was found with a significant increase. The geometric mean of IC50 values reported in 2012 and 2013 were, respectively, 13.84 and 6.484 nM (p < 0.0001) for AMQ; 173.4 and 113.2 nM (p = 0.01) for LUM; 3.322 and 3.673 nM (p = 0.34) for ARS; 39.72 and 18.29 nM (p < 0.0001) for CQ. AMT was only tested in 2013, the geometric mean found was 6.222 nM.

**Table 1:** Ex *vivo* susceptibility of *Plasmodium falciparum* isolates from Thiès in 2012 and 2013

Drug	2012				2013				*p* value
	3D7 IC50 (nM)	Mean with 95 % IC (nM)	Range (nM)		3D7 IC50 (nM)	Mean with 95 % IC (nM)	Range (nM)		
			Min	Max					
							Min	Max	
AMQ	5.488	13.84 (11.84–16.13)	0.7425	63.13	3.321	6.484 (5.208–8.071)	1.925	43.85	<0.0001
LUM	417.5	173.4 (142.0–211.8)	21.26	981.1	164.3	113.2 (85.9–149.1)	17.05	414	0.01
ARS	17.88	3.322 (2.852–3.869)	0.5222	10.16	2.805	3.673 (3.046–4.428)	1.22	18.54	0.34
AMT		–	–	–	7	6.222 (5.013–7.724)	1.188	33.6	–
CQ	17.58	39.72 (30.92–51.02)	2.850	402.9	9.287	18.29 (29.07–87.59)	6.474	384.1	<0.0001

The chloroquine-sensitive strain 3D7 was tested and gave adequate results <100 nM [44]. The difference of values for each molecule for 3D7 was significant excepted for artesunate (p = 0.35). p value indicates the significance of the difference of geometric means of the same molecule between 2012 and 2013

*IC50* 50 % inhibitory concentration, *IC* confidence interval, *Mean* geometric mean, *min* minimum, *max* maximum

### Polymorphism assessment of *pfmdr1* and *pfcrt* genes and haplotype of *mdr1* gene analysis

The 120 and 50 samples collected in Thiès were successfully genotyped. The majority of isolates had the wild type alleles N and K at codon 86 (*pfmdr1*) and 76 (*pfcrt*) with respectively 96 and 78 % in 2012 and 98 and 80 % in 2013. Mixed allele was found in 2012 in 1 % for N86Y and 2 % for K76T of proportion. The mutant allele for these codons was in proportion of 3 and 2 % in 2012 and 2 and 0 % in 2013. Concerning the 184 codon, the mutant allele F predominates both in the 2 years with 75 % for F allele (vs. 20 % for Y allele) in 2012 and 60 % for F allele (vs. 34 % for Y allele) in 2013 with a significant difference (p = 0.029). The presence of mixed allele in Thiès isolates (3 % in 2012 and 6 % in 2013) for this codon was noted. Finally, for 1246 codon, the only allele found was the wild type D1246 (Table 2).

**Table 2:** Prevalence of single nucleotide polymorphism et haplotypes of *pfmdr1* gene of isolates from Thiès

*pfmdr1* genotype	2012 (120)	2013 (N = 50)	p value
N86	97 % (84/87)	98 % (49/50)	0.63
86Y	2 % (2/87)	2 % (1/50)	0.1151
MIXTE (N86Y)	1 % (1/87)	0 %	0.44
Y184	20 % (22/119)	34 % (17/50)	0.029
184F	78 % (93/119)	60 % (30/50)	0.0155
MIXTE (Y184F)	3 % (4/119)	6 % (3/50)	0.42
D1246	100 % (119/119)	100 % (50/50)	–
K76	78 % (47/60)	80 % (40/50)	0.83
76T	20 % (12/60)	20 % (10/50)	1
MIXTE	2 % (1/60)	0 %	0.36
NYD	18 % (18/102)	35.85 % (18/50)	0.012
NFD	79 % (81/102)	62.26 % (31/50)	0.022
YFD	3 % (3/102)	1.89 % (1/50)	0.72

Samples containing single allele in the codons N86Y and Y184F were used to determine prevalence of the circulating *pfmdr1* to reduce ambiguity from the mixed samples. The haplotypes N86Y184D1246, N86F184D1246 and Y86F184D1246 were found both in 2012 and 2013 (Table 2). A high prevalence of N86F184D1246 haplotype was found 79 % in 2012 and 62.26 % in 2013 with a significant decrease in 2013.

Both the wildtype Y184 and the combined N86Y184D1246 are selected in 2013.

### Genotype and phenotype analysis

An association analysis between 184F mutation and the ex vivo susceptibility to LUM, AMT, CQ, AMQ, and ARS was performed (Table 3). High geometric means of IC50 for LUM were found with samples having the wild allele Y184 in 2012 isolates (p = 0.0008). For the other compounds, no difference between the geometric mean of IC50 of isolates with the 184F allele and that of GM of IC50 s with the wild allele was see.

**Table 3:** Association between the mutation 184F and the geometric means of IC50 values for each drugs

Drugs	Year	IC50 geometric mean (nM)		
		Y184	184F	p value
Amodiaquine	2012	10.51 (19)	15.02 (69)	0.0671
	2013	5.1 (16)	6.277 (24)	0.1992
Chloroquine	2012	117.8 (20)	104 (89)	0.1094
	2013	24.18 (15)	28.10 (27)	0.1036
Lumefantrine	2012	493.6 (15)	179.2 (69)	0.0008
	2013	155.9 (15)	68.13 (23)	0.1277
Artesunate	2012	4.034 (12)	3.041 (60)	0.3808
	2013	3.365 (17)	3.693 (25)	0.5729
Artemether	2012	–	–	–
	2013	5.997 (17)	5.936 (25)	0.9387

The numbers in parentheses indicate the number of samples with wild or mutant allele

## Discussion

*Plasmodium falciparum* resistance exists to all first-line anti-malarial drugs, used in both the past and present, alarmingly, even artemisinin derivatives. This resistance has been confirmed in five countries in Southeast Asia (Cambodia, The Lao People’s Democratic Republic, Myanmar, Thailand, Viet Nam) [27]. Monitoring ACT resistance worldwide is absolutely essential, as there is no other alternative drug treatment available [28].

In Senegal, ACT has been first-line treatment since 2006, and currently, AL and ASAQ are recommended as first-line treatment of uncomplicated malaria, while dihydroartemisinin-piperaquine is used as second-line [2, 19]. Both combinations (AL and ASAQ) are effective event for child and adult [29]. However, AL is used more frequently because it is better tolerated [30]. ACT treatment failure of 0.9 % for AL was observed between 2004 and 2014 and it was 0.25 % for ASAQ treatment [2]. Previous studies showed that AL, DHAPQ and ASAQ are highly effective for the treatment of uncomplicated *P. falciparum* malaria in Senegal [31, 32]. In Thiès (2012–2013) three recrudescent samples were observed and these did not reveal mutation in the K13 propeller region (Dieye et al. pers. comm.).

The DAPI ex vivo test, that has been shown to be an excellent technique to study the chemo-sensitivity of *P. falciparum* [20, 25], was used to measure the ex vivo sensitivity of isolates from Thiès in 2012 and 2013 to AMQ, CQ, LUM, ARS, and AMT. Currently, novel phenotype assays (RSA) and a new molecular maker of mutations in the *kelch 13* (K13) propeller region [9], to detect resistance of ART derivatives, are used and recommended [33–35]. However, in this study, the data presented was manipulated in the seasonal period of 2012 and 2013, before the RSA recommendation in December 2013. Kelch 13 sequencing was performed and no mutations known to confer resistance were observed in these samples (Deme & Ndiaye, pers. comm).

To validate the result, 3D7 chloroquine sensitive strain was used. Good sensitivity (<00 nM) was found for chloroquine in both years. A significant increase of sensitivity was observed for 3D7 and isolate from Thiès and this, for all molecules except artesunate, which means the differences in drugs between years could be for technical reasons rather than biological ones. ARS and AMT exert their actions through formation of dihydroartemisinin in vivo [36]. This metabolite is often present in higher concentrations than the parent drugs. However, studies show that AMT and ARS have potent parasite killing activity in vitro [37]. This id why AMT and ARS were tested separately.

Also, five single nucleotide polymorphisms in the *pfmdr1* gene were associated to anti-malarial drug resistance. Studies show that the *pfmdr1* gene polymorphism at codons N86Y, Y184F and D1246Y is mainly linked to AL or ASAQ drug pressure [15, 38, 39]. These facts justify the choice of the three codons of *pfmdr1* gene.

The resistance to AMQ has always been linked to the mutation at the 86 position of the *pfmdr1* gene [10, 11]. Studies show that high ASAQ pressure on the parasite population can select Y86Y184Y1246 haplotypes [15]. The existence of cross resistance, strains resistance to both of drugs together, between AMQ and CQ was detected [40]. The result obtained in Thiès, combined with those obtained in the same region in 2010 [25] and in 2013 [20] reveal that the ex vivo sensitivity of the parasite population to AMQ and CQ has increased between 2011 and 2013. Also, as previously described in Thiès [20], a decrease of the mutation at the 86 position was observed. Studies show that the prevalence of 86Y allele increased between 2000 and 2003 on parasites from Pikine (Dakar). However, a decrease was noted between 2003 and 2009 in this locality [41]. These results reveal that sensitivity of parasite to AMQ increased drastically after the abandonment of chloroquine in 2003. The same tendency was observed in Thiès. Also, for the *Pfmdr1* gene, the haplotype Y86Y184Y1246 was not found. This means that the drugs ARS and AMQ are effective on the population of *P. falciparum* from Thiès and any drug pressure was noted. Increase of wild-type allele K76 was also found between 2011 and 2013 in Thiès. The study of Ndiaye et al. [42] also showed this increase in the central (70.5 % in 2009 and 74.8 % in 2010) and in southern (65.4 % in 2010 and 71.0 % in 2011) Senegal.

The combination AMT-LUM has been used as first-line treatment for uncomplicated malaria in Senegal since 2006 [43]. The resistance to LUM has been linked to the increase of copy number in the *pfmdr1* gene and the selection of the N86 allele [10]. However, studies have shown a selection of the N86F184D1246 haplotype by a high pressure of AL [12, 16]. The high prevalence of wild allele N86, mutant allele 184F and N86F184D1246 haplotype was also noted in this study. In Thiès, Van Tyne et al. [20] demonstrated an increase of the 184F mutation between 2008 and 2011. However, a tendency to a decrease of this mutation between 2011 and 2013 was noted. This tendency can be explained by the use of the ASAQ combination in SLAP (health centre) after the unavailability of AL. This observation means that a pressure is exerted by AMT-LUM combination on the parasite population from Thiès and then this pressure decreases if the drug (AL) is not used.

## Conclusion

The study of ex vivo sensitivity of *P. falciparum* to anti-malarial drugs is a powerful tool to understand parasite phenotypes and to orient the policy of monitoring therapeutic management of malaria. The results of this study show a good sensitivity of *P. falciparum* populations to amodiaquine, artesunate, artemether and lumefantrine. So, the high prevalence of N86F184D1246 haplotype selected by AL pressure was found. Expanded in vivo surveillance of ACT and continued monitoring of artemisinin drug resistance by using new techniques, such as the ex vivo RSA and *kelch13* genotyping, should be a priority.
